# Development of Amyloid PET Analysis Pipeline Using Deep Learning-Based Brain MRI Segmentation—A Comparative Validation Study

**DOI:** 10.3390/diagnostics12030623

**Published:** 2022-03-02

**Authors:** Jiyeon Lee, Seunggyun Ha, Regina E. Y. Kim, Minho Lee, Donghyeon Kim, Hyun Kook Lim

**Affiliations:** 1 Research Institute, Neurophet Inc., Seoul 06234, Korea; jylee@neurophet.com (J.L.); reginaeunyoungkim@neurophet.com (R.E.Y.K.); minho.lee@neurophet.com (M.L.); 2 Division of Nuclear Medicine, Department of Radiology, Seoul St. Mary’s Hospital, College of Medicine, The Catholic University of Korea, Seoul 06591, Korea; seunggyun.ha@gmail.com; 3 Department of Psychiatry, Yeouido St. Mary’s Hospital, College of Medicine, The Catholic University of Korea, Seoul 07345, Korea

**Keywords:** deep learning, PET, MRI, SUVR, amyloid-beta

## Abstract

Amyloid positron emission tomography (PET) scan is clinically essential for the non-invasive assessment of the presence and spatial distribution of amyloid-beta deposition in subjects with cognitive impairment suspected to have been a result of Alzheimer’s disease. Quantitative assessment can enhance the interpretation reliability of PET scan; however, its clinical application has been limited due to the complexity of preprocessing. This study introduces a novel deep-learning-based approach for SUVR quantification that simplifies the preprocessing step and significantly reduces the analysis time. Using two heterogeneous amyloid ligands, our proposed method successfully distinguished standardized uptake value ratio (SUVR) between amyloidosis-positive and negative groups. The proposed method’s intra-class correlation coefficients were 0.97 and 0.99 against PETSurfer and PMOD, respectively. The difference of global SUVRs between the proposed method and PETSurfer or PMOD were 0.04 and −0.02, which are clinically acceptable. The AUC-ROC exceeded 0.95 for three tools in the amyloid positive assessment. Moreover, the proposed method had the fastest processing time and had a low registration failure rate (1%). In conclusion, our proposed method calculates SUVR that is consistent with PETSurfer and PMOD, and has advantages of fast processing time and low registration failure rate. Therefore, PET quantification provided by our proposed method can be used in clinical practice.

## 1. Introduction

Positron emission tomography (PET) neuroimaging tools have been used for in vivo assessment in molecular biology and neuropathology [1]. PET techniques have facilitated early and differential dementia diagnosis, and several PET ligands are available to assess dementia biomarkers and, thus, to support key clinical decision-making. Among these, amyloid PET is widely used to evaluate the spatial distribution of amyloid-beta plaque in patients with cognitive impairment to rule out Alzheimer’s disease (AD) from other dementia diagnosis [2]. Consequently, accurate and effective interpretation of amyloid PET is required, and the demand for automated technology for the interpretation of amyloid PET has increased. An accurate and reliable tool for amyloid PET analysis can advance the research and clinical decision process. Currently, interpretations by nuclear medicine physicians or radiologists constitute the gold standard for amyloid PET, which shows substantial inter-reader agreement of up to 0.8 [3,4]. However, there is room for improvement; one can imagine that the disagreement could be observed with less experienced physicians and/or for equivocal cases. These disagreements could lead to misdiagnosis or delayed clinical decisions. To minimize the rater’s bias and improve the reliability of the amyloid PET interpretation, quantitative evaluation of brain PET has been mostly used in research [5].

There are limiting factors, however, of the use of automated PET analysis tools for clinical purposes. The quantitative measurement of amyloid PET often involves a resource-consuming process (computing power) for 3D image pre-processing, which has been pointed out as a limiting factor. In addition, while there are tools widely used in research (e.g., PMOD and PETSurfer), no study provides a comprehensive analysis of their performance (reliability and validity) in the clinical setting. There are a couple of studies comparing their in-house tools against PMOD. One study compared their in-house method using an atlas-based approach with PMOD and reported faster processing time with possible bias due to the standardization approach [6]. A similar study was also conducted to compare the developed software and PMOD and report interchangeable results with a user-friendly interface [7]. Another atlas-based amyloid PET quantification study reported a high correlation and low average error compared with the magnetic resonance imaging (MRI)-based method but atlas-based methods have a risk of non-optimal calculation in case of severe brain atrophy [8]. Meanwhile, a deep learning-based PET-only quantification method provided a low mean absolute error compared to a MR-based method using FreeSurfer, but the variability of the bias seemed dependent on the magnitude of the amyloid burden [9]. Considering these previous studies, the MR-based method is still most reliable for amyloid PET quantification. For amyloid PET quantification to be introduced into clinical practice, it is necessary to develop a reliable SUVR calculation algorithm while significantly reducing the computing time required for the MR-based method.

In this study, we describe a deep-learning-based approach for automated PET image processing analysis. The processing pipeline has been carefully designed to extract clinically relevant outputs from PET. The proposed approach utilized attention-based U-Net to obtain structural information of MRI for PET images. We hypothesized that the proposed method effectively identifies PET outcomes that are compatible with other tools used in the field, which are PMOD and PETSurfer. The performance of the developed pipeline has been evaluated based on multiple criteria: (1) our in-house dataset was used to compare our performance with existing technologies; (2) an open-source dataset was further utilized to assess the performance to distinguish amyloidosis-positive and negative groups using our proposed approach.

## 2. Materials and Methods

### 2.1. Dataset

#### 2.1.1. In-House Dataset

We used our in-house dataset collected from the Yeouido St. Mary’s Hospital, the Catholic University of Korea. The study involved used 218 subjects with 135 subjects belonging to the amyloidosis-negative group and 83 subjects belonging to the amyloidosis-positive group. Amyloidosis was determined by a nuclear medicine physician with more than 10 years of experience using a visual rating. The dataset was approved by the institutional review board of Yeouido St. Mary’s Hospital, the Catholic University of Korea (no. SC18RNDI0070). The paired flutemetamol PET and MRI was used in this study. The demographic characteristics of the study subject are presented in Table 1.

A dedicated PET/computed tomography (CT) scanner, Biograph 40 TruePoint (Siemens Medical Solutions, Erlangen, Germany) was used to obtain the flutemetamol PET/CT scans. The CT scans were acquired for attenuation correction before the PET scans. Static PET scans were acquired from 90 to 110 min after 185 MBq of flutemetamol injection. Reconstruction of a static image with the 2D-ordered subsets maximization expectation method with two iterations for 21 subsets of reconstruction was applied. The matrix size was 256 × 256, and the voxel size was 1.3364 × 1.3364 × 3 mm${}^{3}$. The MRI was acquired by a 3.0-T scanner MAGETOM Skyra machine equipped with an eight-channel Siemens head coil (Siemens Medical Solutions, Erlangen, Germany). T1-weighted volumetric magnetization-prepared rapid gradient echo scan sequences were obtained with the following parameters: echo time (TE), 2.6 ms; recovery time (TR), 1940 ms; inversion time, 979 ms; field of view (FOV), 230 mm; matrix size, 256 × 256; and voxel size, 1.0 × 1.0 × 1.0 mm${}^{3}$.

#### 2.1.2. Public Dataset: Centiloid Project

In addition, we utilized a public dataset from the Centiloid project [10]. This project aimed to standardize of PET image analysis to reduce issues arising from multiple sites, ligands, acquisition times, reference regions, etc. To this end, the data of 34 subjects of young controls (YC) (under 45 ages) and 47 subjects diagnosed with AD (between 50 and 89 years) from several centers were collected. For the Centiloid projects, the Pittsburgh compound-B (PiB) was injected into the subject, and the image was acquired after 50–70 min. A summary of the Centiloid dataset is also observed in Table 1 and the detailed information about the Centiloid project dataset and demographic information can be found in [10], respectively.

### 2.2. PET Image Interpretation

For the in-house dataset, the flutemetamol PET interpretation was blinded and independent from patient’s clinical information by a nuclear medicine physician. PET images were visually interpreted using a color scale of the rainbow that adjusts to pons activity of 90% maximum intensity and minimum intensity to zero. A binary interpretation was made as positive or negative by comparing the intensities of gray matter and adjacent white matter in five regions of frontal lobes, posterior cingulate and precuneus, lateral temporal lobes, inferolateral parietal lobes, and striatum. If any of those five regions showed iso- or higher uptake intensity in the gray matter than the adjacent white matter, the entire scan was interpreted as a positive scan. A cut-off value for quantitative analysis of SUVR 0.61 was used when the pons as a reference region [11].

### 2.3. Processing Pipeline

**Figure 1:**
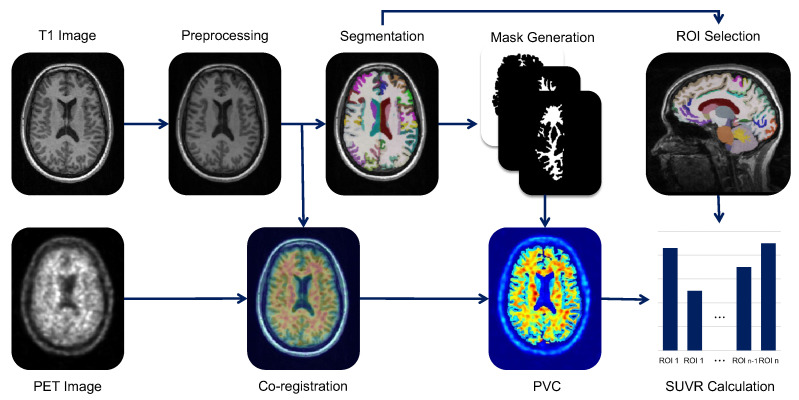
Overview of our proposed PET image processing pipeline. Our proposed pipeline comprises four steps. Step 1 is MRI processing with preprocessing and deep learning-based segmentation. Step 2 is co-registration with preprocessed MRI and PET. Step 3 is partial volume correction (PVC) of PET image. Finally, Step 4 is SUVR quantification from PET.

Our proposed PET processing pipeline comprises four steps: (1) MRI processing, (2) MRI-PET co-registration, (3) partial volume correction, and (4) quantification. Our pipeline is illustrated in Figure 1. We segmented the MRI in individual space using a well-constructed deep learning algorithm. Due to the deep learning segmentation model [12], our approach showed rapid segmentation. Specifically, there is no registration step for the MNI space, namely, standard space mapping for applying a pre-defined brain atlas. Based on this advantage, our pipeline provides an efficient and fast way to fit amyloid PET images.

#### 2.3.1. MRI Preprocessing and Segmentation

We leveraged the structural information based on MRI for PET image quantification and subsequent to analysis. In the previous researches, they analyzed the PET image of the subject by transforming it to the pre-defined general template in the common space [13]; thus, it was time-consuming. To overcome this issue, we used a deep learning network for brain segmentation to obtain the region-of-interest (ROI) in individual space. A U-Net-based model using a split-attention mechanism [12] was employed for brain segmentation in a subject-specific manner. The authors reported the superior performance with efficient running time using the U-Net with region-wise attention module. To apply the image to the network, we performed image to re-sampling and re-orientation with a voxel size of 1 × 1 × 1 mm${}^{3}$ and a right-anterior-superior (RAS) orientation. Using the deep learning model, we obtained 97 ROIs in the brain of each subject.

#### 2.3.2. Co-Registration

For the quantification of the amyloid burden in ROIs based on the MRI, we performed co-registration of PET and MRI. Thus, the uptake volume of amyloid was captured more sensitively by combining the MRI and PET images [14]. For the co-registration, we used the rigid-body linear transformation for PET-MRI registration by minimizing the mutual information based on the method of Mattes et al. [15]. To converge the algorithm, we utilized the gradient descent optimizer with golden-line search methods. In addition, we used linear interpolation for image estimation. By doing this, both the MRI and PET images were positioned in subject-specific space with isotropic volume and RAS orientation. All algorithms were implemented using SimpleITK [16].

#### 2.3.3. Partial Volume Correction

In the PET image, partial volume effect (PVE) due to the poor resolution in limitation of the image scanner was observed [17]. Due to the PVE of the PET images, the intensity of each voxel reflected information for that voxel and neighboring voxels [17,18]. In consequence, the PVE could lead to bias from inaccurate results. To reduce PVE, we conducted partial volume correction (PVC) using a geometric transfer matrix (GTM). We applied GTM to the PET image using the individual ROI mask obtained from the MRI segmentation. For the implementation of PVC, we used the PETPVC toolbox [19]. The point spread function of 2 full-width half-maximum was used to apply the PVC to the in-house dataset. The PVC in our study was not part of the Centiloid dataset.

#### 2.3.4. SUVR Quantification

After PVC, we quantified the PET image based on 97 ROIs from the MRI using the standardized uptake value ratio (SUVR). SUVR is popular for quantifying PET images to compare the inter-subject and intra-subject levels [20]. To measure SUVR, we merged 97 regions into five wide regions (frontal lobes, anterior/posterior cingulate, lateral temporal lobes, lateral parietal lobes, and striatum) according to [21]. Since the number of subregions was different for each of the five regions, we calculated SUVR based on the weighted mean. Additionally, the SUVR was required to reference the region showing a stable degree of PET tracer. In this study, we used the pons as the reference region [11]. Based on these regions, we calculated the SUVR using following the equation [21]:

(1) $${\mathrm{SUVR}}_{n}=\frac{\sum_{n}^{N}\mathrm{MEAN}\left(I_{n}\right)*V_{n}/\sum_{n}^{N}V_{n}}{\mathrm{MEAN}(I_{\mathrm{pons}})},$$

where *N* is the number of ROIs and *I* denotes the intensity of *n*-th ROI. $V_{n}$ is the volume of *n*-th ROI.

### 2.4. Comparative Experiments

To validate our proposed PET processing pipeline, we compared it with the conventional processing tools which are PETSurfer [13,22] and PMOD (PMOD Technologies Ltd., Zürich, Switzerland). Each toolbox was applied to the same in-house dataset. Subsequently, we visually checked the registration and segmentation results for their usability; thus, the images showing poor results were excluded from our analysis.

#### 2.4.1. PETSurfer

PETSurfer is a widely-used open-source software for the analysis of PET images. PETSurfer performs PET processing by utilizing segmentation results from FreeSurfer, involving registration into the MNI space. PETSurfer provides PVC using GTM methods for PVE. After segmentation and registration, PETSurfer automatically calculates the SUVR values with pons as a reference region, volume size in the region, voxel variance, etc. Using the output file of PETSurfer, we calculated the merged five regions with Equation (1).

#### 2.4.2. PMOD

We used the PNEURO tool in PMOD as another comparison method. PMOD’s PNEURO has the following steps for automated PET analysis. First, the MRI is segmented into grey matter, white matter, and cerebrospinal fluid. After generating a tissue probability map, the MRI and PET images are co-registered in the same space. Third, the MRI are spatially normalized to the MNI space to apply them to the pre-defined brain atlas. Subsequently, the PET image is registered using the transform matrix obtained by MRI-MNI registration. We obtained the SUVR results of each ROI from the PET image using the automated anatomical labeling (AAL) atlas. However, the ROI of the AAL atlas was not matched to our ROIs and the PETSurfer ROIs. Therefore, we re-defined the merged five regions to suit the AAL atlas. The defined regions are represented in Table A1. Finally, as with other tools, PVC was also performed with the GTM method in PMOD.

### 2.5. Performance Evaluation

We evaluated the performance of the three tools and compared their results for their reliability and consistency. At first, we compared the SUVR of the positive and negative groups using both the in-house and open-source datasets with *t*-test. Second, we compared the reliabilities across the three tools using intra-class correlation coefficients (ICC) [23,24]. Third, we used the Bland–Altman plot [25] to visualize the degree of agreement between the three tools. Lastly, we conducted the classification using the in-house dataset to compare three tools. In the statistical test, a *p*-value of <0.001 was set to denote statistical significance. All the analyses were performed using R and the Python library. In addition, we reported the processing time and the failure rate of the processing for comparison methods. We calculated the average duration of processing for all subjects to access these validations. Regarding the failure rate of tools, the images were confirmed to evaluate the accuracy visually.

## 3. Results

### 3.1. Subject Demographic

The demographics characteristics of the subjects are shown in Table 1. A total of 218 PET scans paired with T1 MRIs of the subject were analyzed. The amyloidosis positive participants accounted for 38.1% (n = 83) of all the participants. As shown in the Table 1, the APOE ϵ4 and clinical dementia rating were statistically significant. However, there were no statistical differences in education, sex, activities of daily living (ADL), and instrumental activities of daily living (IADL).

### 3.2. SUVR of Amyloid PET from Positive vs. Negative

#### 3.2.1. In-House Dataset Investigation

The comparison of the results of the SUVRs of the amyloidosis-positive and negative groups for the five ROIs and the global region are shown in Table 2. In general, the mean SUVR using our proposed method was between those of PETSurfer and PMOD. For example, the global SUVRs using the proposed method were 0.82 and 0.50 for positive and negative groups, respectively, which were higher than PETSurfer (0.78, positive; 0.45, negative) and lower than those of PMOD (0.84, positive; 0.52, negative). The trend cut across all the five sub-structures we investigated including frontal, anterior-posterior cingulate, lateral parietal, lateral temporal, and striatum. We also used *t*-test to compare the SUVR measurements by the three for any statistically significant mean difference for the clinical groups in general population. As shown in the table, we observed that all three methods successfully identified significant differences across the two clinical groups (*p*-value < 0.001).

#### 3.2.2. Centiloid Dataset Investigation

To validate the performance of our proposed approach, we additionally compared SUVR between positive and negative groups using the Centiloid dataset. The results are shown in Table 3. Similar to the amyloidosis group results above, the SUVR obtained from our pipeline showed a significant difference (p< 0.001) between the AD and YC groups for all five subregions and global measures.

### 3.3. SUVR Correspondence between the Proposed Method, PMOD, and PETSurfer

**Figure 2:**
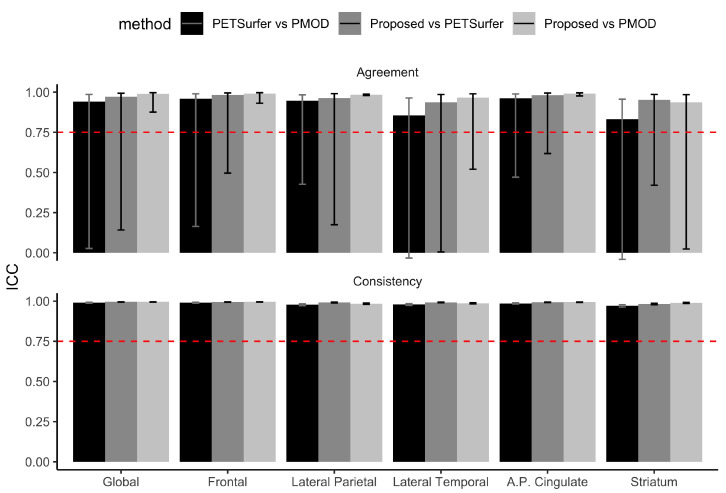
Intraclass correlation for PETSurfer, PMOD, and the proposed method. Dashed line shows that the ICC value is 0.75.

The correspondence between three technologies was compared using ICC and Bland–Altman plots for their reliability and agreements. The 218 in-house data reported in the previous section were utilized, and the results were described as follows:

**Intra-class correlations coefficient:** The ICC results are reported in Figure 2 and Table 4. All the comparative results for the three tools showed excellent ICC as shown in Table 4 and based on criteria suggested in [26] (>0.9, excellent). Specifically, all the regions showed ICC values greater than 0.9 except for the lateral temporal lobe (ICC = 0.86) the striatum (ICC = 0.82) between PETSurfer and PMOD.

**Figure 3:**
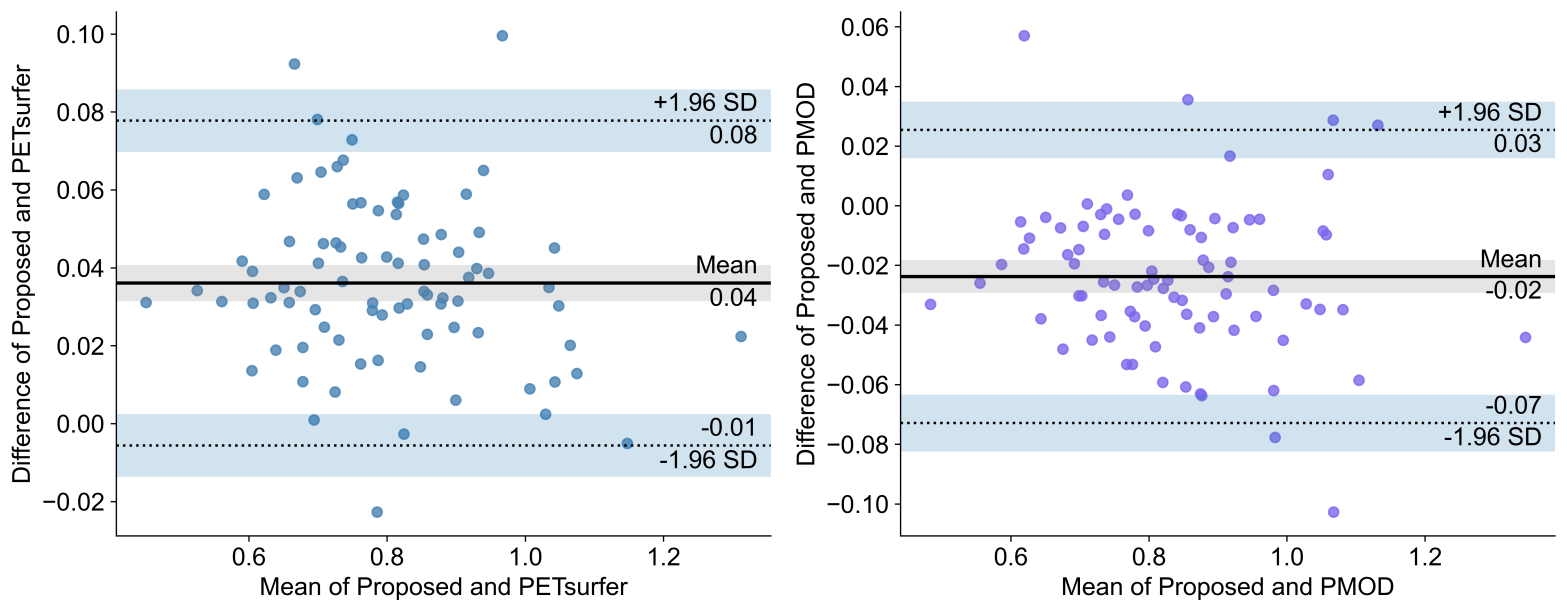
Bland–Altman plot of the proposed method, PETSurfer, and PMOD for the global region of amyloidosis-positive subjects.

**Figure 4:**
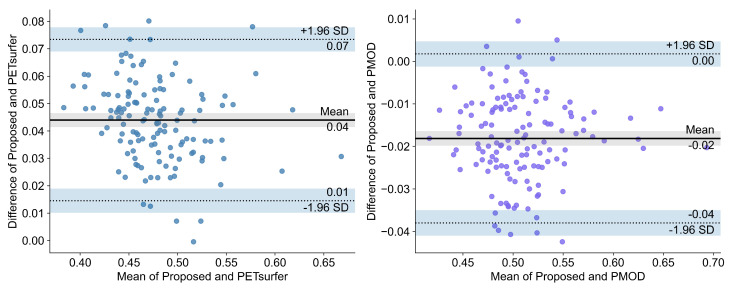
Bland–Altman plot of the proposed method, PETSurfer, and PMOD in the global region of amyloidosis-negative subjects.

**Figure A1:**
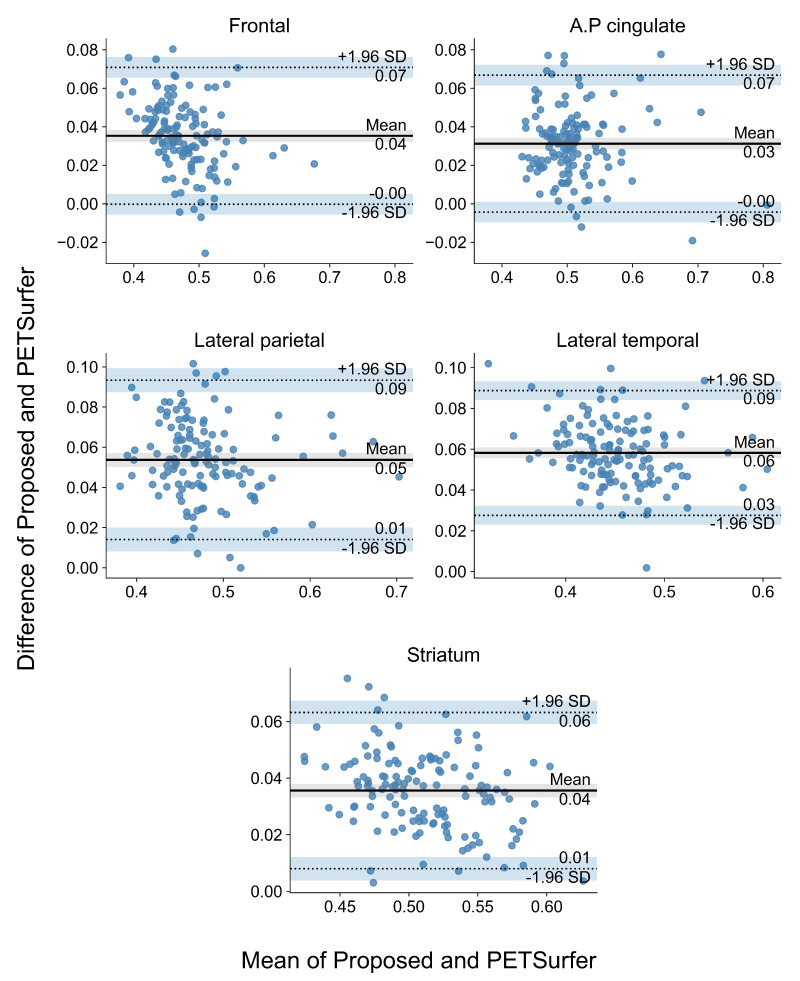
Bland–Altman plot for amyloidosis-negative subject for the comparison of our proposed method and PETSurfer. A.P. cigulate denotes anterior posterior cingulate regions.

**Figure A2:**
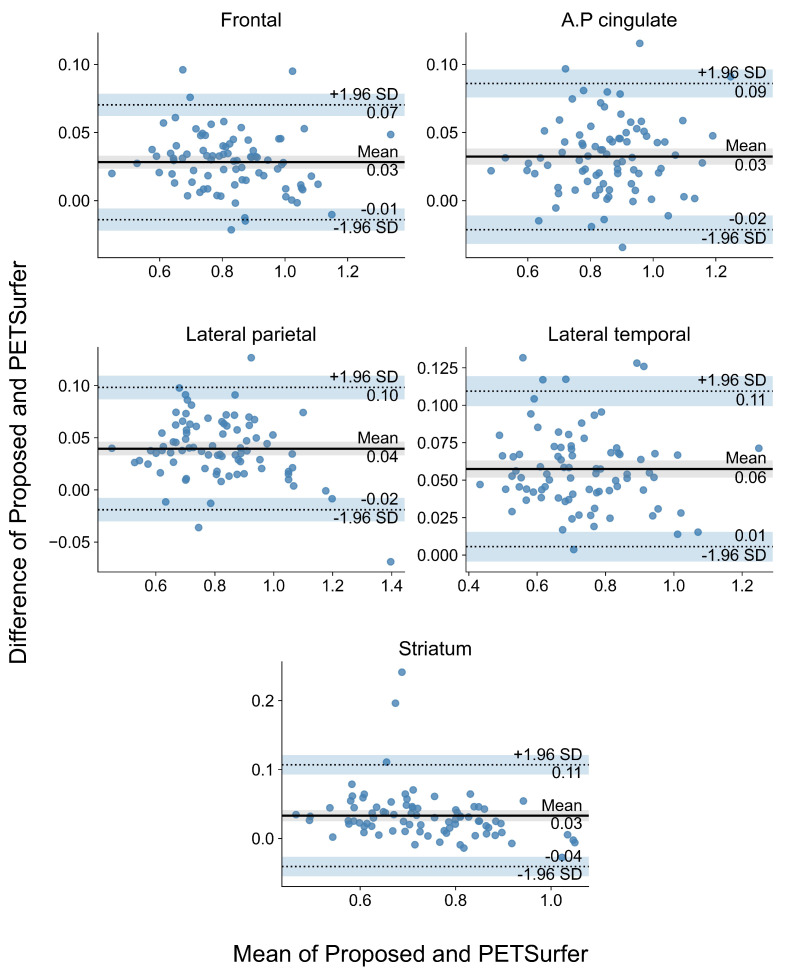
Bland–Altman plot for amyloidosis-positive subject for the comparison of our proposed method and PETSurfer. A.P. cigulate denotes anterior posterior cingulate regions.

**Figure A3:**
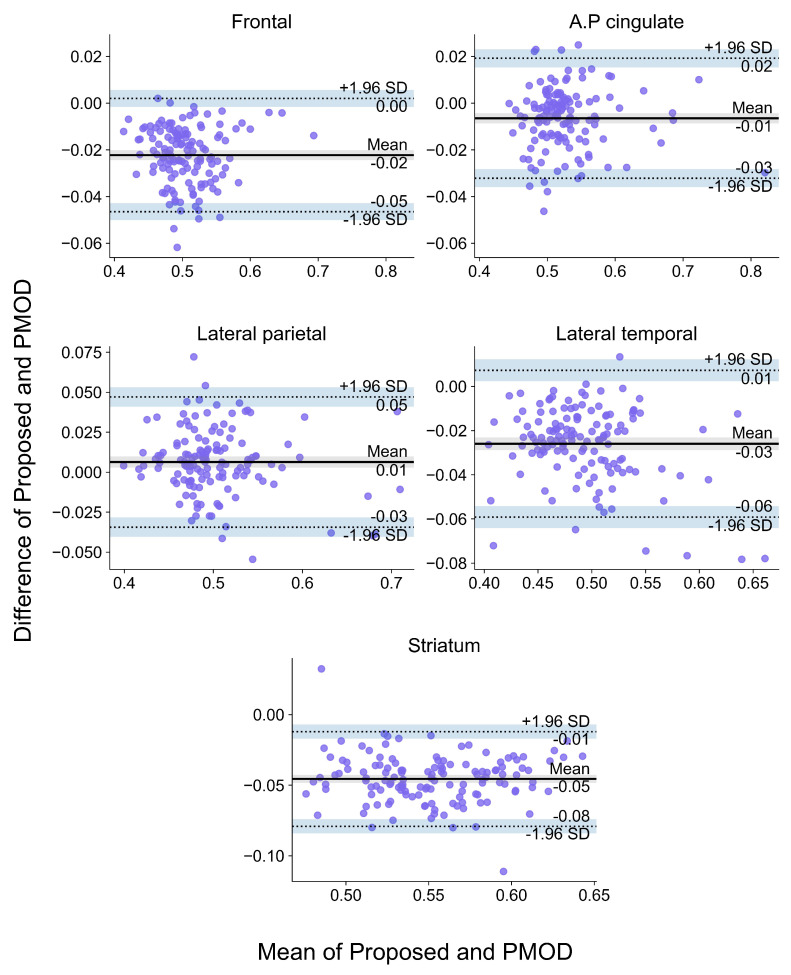
Bland–Altman plot in amyloidosis-negative subject for the comparison of our proposed method and PMOD. A.P. cigulate denotes anterior posterior cingulate regions.

**Figure A4:**
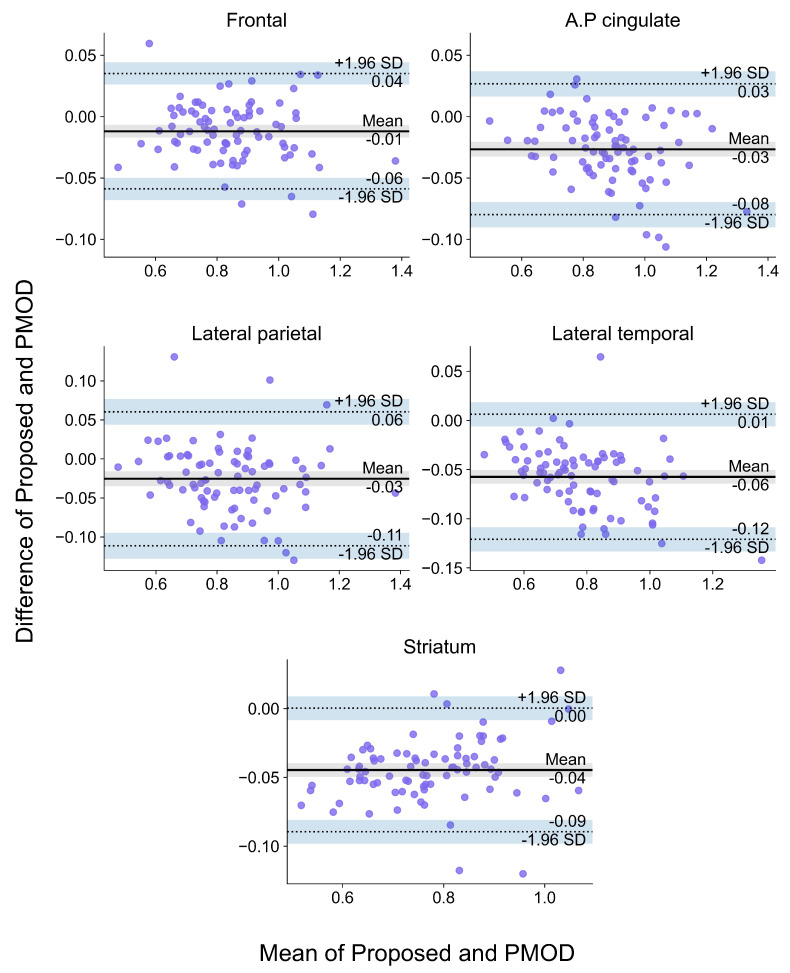
Bland–Altman plot in amyloidosis-positive subject for the comparison of our proposed method and PMOD. A.P. cigulate denotes anterior posterior cingulate regions.

**Bland–Altman plot:** The Bland–Altman plots are visualized in Figure 3 and Figure 4. As shown on the left of Figure 3 for negative subjects, the mean difference between the proposed method and PETSurfer was 0.04 (95% limits of agreement; LOA: −0.01, 0.08). For the results of the proposed method and PMOD, the mean difference was −0.02 (95% LOA: −0.07, 0.03). Regarding the positive subjects in Figure 4, the mean difference between the proposed method and PETSurfer in the left plot of the figure is 0.04 (95% LOA: 0.01, 0.07). On the other hand, the mean difference between the proposed method and PMOD is −0.02 (95% LOA: −0.04, 0.00). All regions of the Bland–Altman plot are represented in Figure A1, Figure A2, Figure A3 and Figure A4.

### 3.4. Amyloid Classification with Cut-Off Value

We carried out the classification using the cut-off value (>0.61) based on the pons as a reference region to evaluate the ability to identify the subject of our proposed pipeline. To perform the classification, we defined the target and prediction labels using our in-house dataset. For the prediction label, if the global SUVR was higher than 0.61, we set the label as 1. By contrast, if the global SUVR was lower than 0.61, we set the label as 0. For the ground truth (target label), we used mentioned earlier binary visual scoring results by the expert. Based on this, we calculated the AUC, accuracy (ACC), sensitivity (SEN), and specificity (SPEC). The results are represented in Table 5. As shown in the table, we observed that the SUVR value for each method was precisely identified between two groups (positive vs. negative) by achieving the promised performance in four metrics. Specifically, our proposed pipeline and PMOD showed a higher specificity than sensitivity. Unlike our results and those of PMOD, PETSurfer showed a the higher sensitivity than specificity. In addition, the AUCs of the three tools were above 0.95 and similar.

*Processing Time and Failure Rate*

The processing time was determined based on the average time subject-by-subject. The results are summarized in Table 6:

PMOD and PETSurfer take approximately 45.7 and 23.5 min to perform registration, segmentation, and PVC. Of note, the time taken by PETSurfer was used only for PET image processing. PETSurfer was required Freesurfer before processing. It could take about 12 h. By contrast, our proposed method process takes approximately 14.8 min. It is faster than other tools.

The processing failure rate was calculated based on the visual check. Specifically, we confirmed how well-paired images co-registered regardless of comparative tools. First, we counted the completely failed registration pair. Second, we divided the failure case subject for each tool. Lastly, each tool’s failure rates were calculated for the entire dataset. The results are represented in Table 6. For our proposed pipeline, the image of a subject was not registered, thus the failure rate was 1.2% (3/239). For PETSurfer, the failure rate was 0.4% (1/239). For PMOD, the failure rate was higher than those of other comparative tools; the images of 21 subject were not registered. The failure rate of PMOD was 8.7% (21/239).

## 4. Discussion

In this study, we described a deep-learning-based approach for PET image analysis and compared its performance with those of two well-known tools in the field, PETSurfer and PMOD. Our investigation showed a high correspondence between our proposed method and the two other methods in computing SUVR values for flutometamol for the general population and for Pittsburgh compound-B for the Centiloid dataset. The correspondence of our proposed method to PMOD was higher than that to PETSurfer in general, though the overall classification accuracies were similar. The Bland–Altman plot showed that our measurements were in reasonable agreement. Our investigation indicated that our proposed method can evaluate clinically relevant SUVR values and is compatible with other widely used tools in the field.

Our ICC investigation showed excellent agreement across the three methods in general. The ICC greater than 0.75 was a bottom cutoff suggested by Shrout and Fleiss [24] for two raters are on agreement. Our results indicated that any two methods were in good agreement as suggested by Shrout and Fleiss [24]. Among those, the striatum and lateral temporal regions presented a slightly lower agreement and reliability than PETSurfer and PMOD than other results (still much higher than 0.75). These relatively lower ICC values in the striatum can be attributed to several factors. One factor is the imperfect segmentation of the striatum. Since the striatum consists of putamen and caudate, which is a mixture of white and gray matter, segmentation can be very challenging. In the previous literature, the study reported that the SUVR for the deep brain structures, e.g., the caudate and putamen, were easily affected by the quality of the segmentation algorithm than the cortical regions [27]. The other previously published study evaluated that the PVC results are dependent on the segmentation results for structure information from MRI [28]. Another explanation is that it is more difficult for flutemetamol to detect the amyloid burden than the other ligand [29] in the striatum. This may also be the reason for the lower ICC in the striatum. Regarding the lateral temporal regions, atrophy could cause the lower ICC in this region due to the smaller size in the lateral temporal lobe. According to the previous report [30,31], the atrophy could result in inaccurate SUVR measurement by PVE. Because part of our dataset includes memory deficit subjects with older age, excessive atrophy might present in their brain. Additionally, the lateral temporal consisted of a few regions, i.e., middle temporal and superior temporal. Since we calculated SUVR using the weighted mean of those sub-regions, regions larger than lateral temporal area, which includes more than two sub-regions, might be relaxed from the effect of atrophy by merging more regions. Further studies are required for better understanding.

The Bland–Altman plot showed that the mean biases of our proposed pipeline with PETSurfer and PMOD are only 0.04 and −0.02, respectively, for both amyloidosis-positive and negative groups. Accordingly the absolute systematic error of the proposed method with PETSurfer and PMOD is very small. The variability of bias is not dependent on the magnitude of the amyloid burden. Therefore, the agreement of our method with PETSurfer and PMOD is excellent. The SUVR values for the proposed method are almost interchangeable with SUVR values from PMOD and PETSurfer, considering the very small bias and similar classification performance with the same cutoffs between our proposed method and PMOD or PETSurfer.

Registration has been considered an important factor in having a precise SUVR [32]. To minimize effect of mis-registration for the comparison of SUVR across three tools, we visually confirmed the registration results. We observed that PMOD had a higher failure rate in registration than other tools, which were removed from our comparative study. Please note that, while PMOD provides additional step solutions for re-aligning the failed registration cases, such as the manual align option, the re-aligning was not tried in this study.

Our proposed PET pipeline is faster than other toolboxes in the filed. Our study demonstrates replaceable performance of our proposed method for clinical use. When compared to other approaches, one main difference of our proposed method is the involvement of the deep network approach for brain segmentation, which performed in one’s original space. The deep-learning-based segmentation showed enhanced speed and comparable results. In contrast, two previously developed technologies, i.e., PMOD and PETSurfer, utilized the conventional segmentation method (voxel-based and surface-based). Additional mapping to common space, which is the MNI space, for matching the pre-defined atlas or regions is not required in our process. It can be inferred that individual-based segmentation has fewer spatial deformations than conventional methods and is cost-effective.

Furthermore, our proposed deep-learning-based pipeline does not require additional 3D image processing. We engineered our segmentation pipeline utilizing deep learning methods without any pre-processing, such as noise reduction and/or probability map generation. Our pipeline without pre-processing steps reduces the processing time and required resources and, thus, efficiently applicable to the conventional 3D PET imaging analysis with less cost.

## 5. Conclusions

Brain amyloid PET has been widely used to diagnose Alzheimer’s disease. To better quantify the PET imaging outcomes in clinical settings, several automated processing toolboxes were proposed. There is, however, still room for improvement, specifically regarding the amount of resources needed for PET analysis. Therefore, to efficiently process the PET images, we proposed a novel deep-learning-based PET analysis pipeline. In particular, we leveraged the deep learning network for MRI brain segmentation. Then, co-registered PET image was used to calculate the SUVR. The novel deep-learning-based approach for quantitative analysis of amyloid PET imaging demonstrates reasonable performance comparable to those of PETSurfer and PMOD. Our proposed pipeline can be used in the clinical setting, with its reliable SUVR calculation, high classification accuracy, and faster computation time. Our automated PET quantification method is expected to help the clinical decision by providing reliable outcomes for PET in the relative field.

## Figures and Tables

**Table 1:** The demographics of the amyloidosis-positive and negative groups. Mean (standard deviation) or percentage with the number of the subject N are reported. *p*-value was calculated from the *t*-test between two groups of in-house dataset. Note that the two subjects of YC and one subjects of AD in Centoloid dataset refused the APOE gene test. In the clinical dementia rating score of Centoloid dataset, the AD subject was in range of 0.5–1.

	Amyloidosis Group			Centiloid Dataset	
	Negative	Positive		YC	AD
	(N = 135)	(N = 83)	*p*-Value	(N = 34)	(N = 47)
Age, years	71.9 ± 10.4	77.1 ± 6.9	<0.001	31.5 ± 6.3	67.5 ± 10.5
Female, %(N)	73.3 (99)	67.5 (56)	0.439		
APOE ϵ4 carriers, %(N)	16.3 (22)	47.0 (39)	<0.001	25.0 (8)	64.0 (28)
Clinical Dementia Rating sum of Box	1.4 ± 1.9	2.7 ± 3.0	0.001		
Clinical Dementia Rating	0.3 ± 0.3	0.6 ± 0.5	<0.001		0.5–1
Education, years	11.0 ± 4.7	10.9 ± 5.1	0.911		
ADL	0.5 ± 1.8	0.9 ± 3.3	0.380		
IADL	5.5 ± 7.2	9.3 ± 8.8	0.002		

*Positive and negative*, amyloidosis existent and no-existent group. *p*-value, *t*-test or *χ*^2^ test for positive and negative groups, where applicable.

**Table 2:** Mean SUVR and standard deviation for the three tools (Proposed, PETSurfer, and PMOD) are summarized for 218 participants using flutometamol PET scans.

	Proposed				PETSurfer				PMOD			
	Positive	Negative	*p*-Value		Positive	Negative	*p*-Value		Positive	Negative	*p*-Value	
Frontal	0.84 ± 0.15	0.49 ± 0.04	<0.001		0.81 ± 0.16	0.46 ± 0.05	<0.001		0.85 ± 0.15	0.56 ± 0.04	<0.001	
A.P. cingulate	0.87 ± 0.15	0.52 ± 0.05	<0.001		0.84 ± 0.14	0.49 ± 0.05	<0.001		0.90 ± 0.15	0.53 ± 0.05	<0.001	
Lateral parietal	0.83 ± 0.18	0.50 ± 0.05	<0.001		0.79 ± 0.17	0.45 ± 0.05	<0.001		0.86 ± 0.17	0.50 ± 0.06	<0.001	
Lateral temporal	0.76 ± 0.15	0.47 ± 0.04	<0.001		0.70 ± 0.15	0.42 ± 0.05	<0.001		0.81 ± 0.16	0.51 ± 0.05	<0.001	
Striatum	0.75 ± 0.12	0.53 ± 0.04	<0.001		0.72 ± 0.14	0.49 ± 0.04	<0.001		0.79 ± 0.12	0.57 ± 0.04	<0.001	
Global	0.82 ± 0.15	0.50 ± 0.04	<0.001		0.78 ± 0.15	0.45 ± 0.05	<0.001		0.84 ± 0.15	0.52 ± 0.04	<0.001	

Positive, amyloidosis existent group; Negative, denotes no amyloidosis group; A.P., Anterior/posterior. *p*-value, *t*-test between positive and negative groups of amyloidosis.

**Table 3:** Averaged SUVR of the Centiloid dataset with *p*-value.

	YC	AD	*p*-Value
Frontal	0.66 ± 0.06	1.28 ± 0.14	<0.001
A.P. cingulate	0.74 ± 0.07	1.38 ± 0.12	<0.001
Lateral parietal	0.68 ± 0.06	1.27 ± 0.15	<0.001
Lateral temporal	0.63 ± 0.06	1.19 ± 0.16	<0.001
Striatum	0.74 ± 0.05	1.24 ± 0.14	<0.001
Global	0.67 ± 0.06	1.26 ± 0.14	<0.001

A.P. denotes anterior posterior. AD is Alzheimer’s disease. YC is young control.

**Table 4:** Intra-class correlation coefficient (ICC) for three methods, PETSurfer, PMOD, and our proposed method. The paired ICC values are shown.

		Absolute			Consistency		
	ROI	ICC	(L,	U)	ICC	(L,	U)
PETSurfer vs. PMOD	Global	0.94	(0.03,	0.99)	0.99	(0.99,	0.99)
	Frontal	0.96	(0.16,	0.99)	0.99	(0.99,	0.99)
	Lateral Parietal	0.95	(0.43,	0.98)	0.98	(0.97,	0.98)
	Lateral Temporal	0.86	(−0.03,	0.96)	0.98	(0.97,	0.98)
	A.P. Cingulate	0.96	(0.47,	0.99)	0.99	(0.98,	0.99)
	Striatum	0.83	(−0.04,	0.96)	0.97	(0.96,	0.98)
Proposed vs. PETSurfer	Global	0.97	(0.14,	0.99)	1.00	(0.99,	1.00)
	Frontal	0.98	(0.50,	1.00)	1.00	(0.99,	1.00)
	Lateral Parietal	0.96	(0.17,	0.99)	0.99	(0.99,	0.99)
	Lateral Temporal	0.94	(0.01,	0.99)	0.99	(0.99,	0.99)
	A.P. Cingulate	0.98	(0.62,	0.99)	0.99	(0.99,	1.00)
	Striatum	0.95	(0.42,	0.99)	0.98	(0.98,	0.99)
Proposed vs. PMOD	Global	0.99	(0.88,	1.00)	1.00	(0.99,	1.00)
	Frontal	0.99	(0.93,	1.00)	1.00	(0.99,	1.00)
	Lateral Parietal	0.98	(0.98,	0.99)	0.98	(0.98,	0.99)
	Lateral Temporal	0.96	(0.52,	0.99)	0.99	(0.98,	0.99)
	A.P. Cingulate	0.99	(0.98,	1.00)	0.99	(0.99,	1.00)
	Striatum.	0.94	(0.02,	0.98)	0.99	(0.99,	0.99)

**Table 5:** Classification results of three tools. ACC, SEN, and SPEC denote accuracy, sensitivity, and specificity, respectively.

	AUC	ACC	SEN	SPEC
Proposed	0.9593	0.9633	0.9412	0.9774
PETSurfer	0.9652	0.9587	0.9868	0.9437
PMOD	0.9553	0.9587	0.9405	0.9701

**Table 6:** Results of processing time and failure rate.

	Processing Time	Failure Rate (%)
Proposed	14.8 min ± 1.0 min	1.2
PMOD	45.7 min ± 4.3 min	8.7
PETSurfer	23.5 min ± 3.0 min	0.4

## Data Availability

Not applicable.

## Appendix A. Region Information

**Table A1:** ROI comparison between Freesurfer and AAL at the lobe level. rh and lh denotes the right hemisphere and left hemisphere, respectively. A.P. cigulate means anterior posterior cingulate.

	Freesurfer	AAL
Frontal	ctx-lh/rh-caudalmiddlefrontal	Superior frontal gyrus, dorsolateral (left/right)
	ctx-lh/rh-lateralorbitofrontal	Superior frontal gyrus, orbital part (left/right)
	ctx-lh/rh-medialorbitofrontal	Middle frontal gyrus (left/right)
	ctx-lh/rh-parsopercularis	Middle frontal gyrus orbital part (left/right)
	ctx-lh/rh-parsorbitalis	Inferior frontal gyrus, opercular part (left/right)
	ctx-lh/rh-parstriangularis	Inferior frontal gyrus, orbital part (left/right)
	ctx-lh/rh-rostralmiddlefrontal	Rolandic operculum (left/right)
	ctx-lh/rh-superiorfrontal	Supplementary motor area (left/right)
	ctx-lh/rh-frontalpole	Olfactory cortex (left/right)
		Superior frontal gyrus, medial (left/right)
		Superior frontal gyrus, medial orbital (left/right)
		Gyrus rectus (left/right)
A. P. cingulate	ctx-lh/rh-caudalanteriorcingulate	Anterior cingulate and paracingulate gyri (left/right)
	ctx-lh/rh-isthmuscingulate	Median cingulate and paracingulate gyri (left/right)
	ctx-lh/rh-posteriorcingulate	Posterior cingulate gyrus (left/right)
	ctx-lh/rh-rostralanteriorcingulate	
Lateral parietal	ctx-lh/rh-inferiorparietal	Superior parietal gyrus (left/right)
	ctx-lh/rh-precuneus	Inferior parietal, but supramarginal and angular gyri (left/right)
	ctx-lh/rh-superiorparietal	Supramarginal gyrus (left/right)
	ctx-lh/rh-supramarginal	Angular gyrus (left/right)
		Precuneus (left/right)
Lateral temporal	ctx-lh/rh-middletemporal	Superior temporal gyrus (left/right)
	ctx-lh/rh-superiortemporal	Middle temporal gyrus (left/right)
Striatum	subctx_lh/rh_caudate	Caudate nucleus (left/right)
	subctx_lh/rh_putamen	Lenticular nucleus, putamen (left/right)
